# Off-label and investigational drugs in the treatment of gambling disorder: a critical review

**DOI:** 10.3389/fphar.2026.1806954

**Published:** 2026-05-01

**Authors:** Leon Bissig, Jochen Mutschler

**Affiliations:** Psychiatric Services Lucerne, Lucerne, Switzerland

**Keywords:** behavioral addiction, gambling disorder, investigational, off-label, pathological gambling, pharmacological treatment

## Abstract

Gambling disorder or pathological gambling is a psychiatric disorder with severe and wide-ranging consequences for the affected individual and their surroundings. While no pharmacological treatment has yet been officially approved for treatment, various drugs have been investigated to date. An electronic literature search was conducted in PubMed to identify open-label trials, randomized controlled trials, meta-analyses, other reviews and case reports. In addition, the ClinicalTrials.gov registry was reviewed to identify recent advancements, investigational drug candidates, and ongoing or forthcoming clinical research related to this topic. In this review we summarize and compare Fluvoxamine, Paroxetine, Sertraline, Citalopram, Bupropion, Nefazodone, Naltrexone, Nalmefene, Naloxone, Lithium, Valproate, Topiramate, Olanzapine, N-acetylcysteine, Disulfiram, Acamprosate, Memantine, Amantadine, Gabapentinoids, Modafinil, GLP-1 receptor agonists and Ketamine/Esketamine. From the above-mentioned substances, many have shown promising results in the treatment of gambling disorder, particularly opioid antagonists. However, the small number of studies on the respective drugs, as well as inconsistent designs and contradictory results, limit the significance and clinical applicability of the findings. Here we discuss the most promising drugs, neurobiological pathomechanisms of gambling disorder and potential methodological designs for future studies on this topic.

## 1 Introduction

Gambling is a widespread and common recreational activity in most countries and cultures today. A recent systematic review and meta-analysis found that 46.2% of adults and 17.9% of adolescents worldwide have gambled in the past 12 months (Tran et al., 2024). The prevalence of gambling disorder (GD) in the US and Europe is estimated to be between 0.42% and 1.3% (Loo et al., 2019; Gabellini et al., 2023). GD is characterized by persistent and recurrent gambling behavior, which leads to significant impairment or distress. It has serious and wide-ranging consequences for the affected individual, their surroundings and for society as a whole. Most frequent among these are financial debt, which can lead to criminal activities, psychiatric comorbidity, impaired quality of life and destruction of family ties (Bergh and Kühlhorn, 1994; Ladouceur et al., 1994; Shaw et al., 2007; Lorenz and Shuttlesworth, 1983). Especially concerning is the high rate of suicidality associated with GD. Meta-analytic data show that about one-third of people with gambling problems have suicidal thoughts during their lifetime, and 13.2% have attempted suicide at least once (Kristensen et al., 2024). Among individuals with GD who seek treatment, suicide rates are even higher, further highlighting the devastating impact of this disorder (Battersby et al., 2006; Ronzitti et al., 2017). Gambling is not limited to card games or casinos: there are many forms, including sports betting, loot boxes in gaming, lottery tickets, stock and crypto trading, and much more. With recently launched online prediction markets such as Polymarket, people can now bet on almost anything, such as weather conditions, election results, or whether two countries will go to war within a certain period of time. Gambling is also aggressively promoted to an increasingly younger generation via social media and influencers (Nordin and Eklundh, 1999; Carrasco et al., 1994), so it is likely to become an ever-greater problem in the future. Cognitive behavioral therapy is considered an important and effective form of treatment for GD (Cowlishaw et al., 2012; Jiménez-Murcia et al., 2007). However, not all individuals with GD achieve successful results with CBT alone, so additional treatment options remain an open question. No medication has been officially approved for the treatment of GD, although many pharmacological agents have been investigated in recent decades. To date, the use of medication remains an off-label and experimental treatment option for GD, so their use depends mainly on the physician’s experience. This article focuses primarily on summarizing and contextualizing existing studies on the effectiveness of various drugs in the treatment of GD with the goal of broadening clinician’s views on their potential use cases.

## 2 Methods

This review is intended to provide an overview of the existing open-label and randomized controlled trials on the predominantly pharmacological treatment of GD with a focus on their respective strengths and limitations. In addition, case reports, more recent meta-analyses and other review articles were also included. Since no medication has yet been officially approved for the treatment of GD, the aim is to evaluate which medication could be used for different patient groups and which of the numerous medications warrant further research.

This paper is based on detailed literature analysis and data extraction from the PubMed database, focusing on existing publications regarding the use of off-label pharmacologic agents in the management of GD. In addition, the ClinicalTrials.gov registry was reviewed to identify recent advancements, investigational drug candidates, and ongoing or forthcoming clinical research related to this topic. The following key words were used in literature research of PubMed: gambling disorder, pathological gambling, gambling treatment, behavioral addiction, pharmacological treatment, off-label and investigational use. These terms were used in combination with the different medications discussed in this paper. Approximately 850 papers were found and reviewed. 35 open-label and randomized placebo-controlled trials investigating the efficacy of the respective medications in GD were evaluated.

## 3 Drugs

### 3.1 Selective serotonin reuptake inhibitors

Selective serotonin reuptake inhibitors (SSRIs) are used for the treatment and prevention of new episodes of major depressive disorder, anxiety disorders, posttraumatic stress disorder, and obsessive-compulsive disorder (OCD) among others (Khouzam et al., 2003). Although GD currently is not classified within the OCD-spectrum, there are phenomenological similarities between them (Black et al., 2010). DSM-IV and ICD-10 list pathological gambling (PG) as an impulse control disorder (although DSM-V now lists it as a substance related and addictive disorder) which many of can be treated successfully with SSRIs. SSRIs act by inhibiting the presynaptic serotonin transporter (SERT), thereby enhancing serotonergic neurotransmission (Benmansour et al., 1999). Evidence from multiple studies indicates that serotonergic signaling is altered and dysfunctional in individuals with GD, suggesting a potential therapeutic role for SSRIs in its treatment (Nordin and Eklundh, 1999; Carrasco et al., 1994; Zakeri and N Potenza, 2012).

Bechara et al. employed the Iowa Gambling Task to demonstrate that patients with lesions in the ventromedial prefrontal cortex exhibited an inability to resist immediate rewards, despite evident long-term negative consequences (Bechara, 2003). This behavioral pattern can also be observed in individuals with GD as well as individuals with substance use disorders and suggests possible dysfunction in brain regions associated with reward processing and decision-making. By stimulating the serotonergic system with an SSRI, the participants showed improvements in conscious decision-making regarding more advantageous long-term outcomes during the task. To date, several SSRIs have been evaluated in clinical studies for their efficacy in treating GD, with varying degrees of success.

#### 3.1.1 Fluvoxamine

Hollander et al. (1998) demonstrated promising outcomes in a short-term, single-blind study, where 70% of 10 completers achieved full abstinence from gambling and clinically significant reductions in symptom severity, measured via improvements in clinician-rated CGI scores (PG-CGI-I) and decreases in Yale-Brown obsessive compulsive scale modified for PG (PG-YBOCS) (Hollander et al., 1998). A subsequent 16-week randomized, double-blind crossover trial by the same authors found significant improvement on the PG-CGI scale for fluvoxamine (mean dose at end point 195±50 mg/day) over placebo (40.6% vs. 16.6%, p < 0.005), although PG-YBOCS improvements did not reach statistical significance (Hollander et al., 2000). A post-hoc analysis revealed continued efficacy of fluvoxamine during the second treatment phase, suggesting a diminishing placebo effect over time. While this study showed promising results, it was limited by a small, male-only sample size with a lack of subject heterogeneity and follow-up time.

Blanco et al. conducted another double-blind placebo-controlled study in 2002 in which 32 participants were treated for 6 months with fluvoxamine 200 mg/day at end point. In terms of time and money spent on gambling per week, no statistically significant differences were found between fluvoxamine and placebo in the overall sample, but fluvoxamine was found to be significantly superior in men and young patients (Blanco et al., 2002). In addition, the number of responders to fluvoxamine was found to increase over time, while the number of responders in the placebo group decreased over time, which is consistent with the findings of Hollander et al. and may be further evidence of fluvoxamine’s efficacy. However, it should be noted that only 40% of participants completed the study. The authors concluded that the lack of compliance and retention of participants is one of the biggest challenges when treating or conducting trials with individuals with GD. Another recurring limiting factor was the high response rate to placebo (59%), which was almost twice as high as expected by the authors, again making it difficult to detect statistically significant differences.

In a randomized blind-rater study from 2005, Dannon et al. compared topiramate with fluvoxamine and came to similar results as Hollander et al. did in 1998, namely that completers on Fluvoxamine achieved complete or partial remission of gambling behavior (Dannon et al., 2005a). It also came with similar limitations including a lack of a placebo control group, a small and homogenous sample size and a short period of administration of only 12 weeks. The authors proposed that from their clinical experience individuals with GD exist along a continuum between impulsivity and obsessive-compulsiveness, and that SSRIs like fluvoxamine may be particularly beneficial for individuals with predominant obsessive-compulsive symptoms. This thesis is supported by Hollander et al. which concluded that medication should oftentimes be adjusted to the comorbidity and that, for example, fluvoxamine could be most effective in patients with OCD.

In one of the few follow-up studies on this matter, Dannon et al. assessed relapse rates after drug discontinuation. Among six fluvoxamine responders, 50% relapsed within 6 months, although gambling losses remained lower than baseline (Dannon et al., 2007). The authors hypothesized that the medication could have caused lasting changes in the neurotransmitter systems of the central nervous system and/or in the neuromotivational pathways during these 6 months, overall decreasing the chances of a relapse. They also suggested that a longer period of reduced or abstinent gambling behavior may have led to cognitive behavioral changes in the test subjects, additionally lowering the relapse risk. The absence of significant differences between fluvoxamine and other agents (topiramate, bupropion, naltrexone) and lack of placebo control limit interpretability. The authors also pointed to a study by Slutske in which she estimates that about one-third of all individuals with GD in the U.S. eventually recover naturally without any treatment, which in turn raises further questions about the need for or efficacy of medication in the long-term treatment of GD (Slutske, 2006).

Overall, the available studies support the use of fluvoxamine in male individuals with GD, at least in an acute phase of 8–12 weeks of treatment. Based on numerous clinical experiences suggesting the existence of subtypes in the gambling group, it is reasonable to speculate that fluvoxamine and other SSRIs may be most effective in patients with prevalent obsessive thoughts and compulsive behaviors or co-occurring depressive disorder. Further studies investigating the effect of different drugs on specific patient subgroups would be necessary to investigate this hypothesis in more detail. In addition, more placebo-controlled studies would be needed, which would have to run over a longer period of time in order to be able to make statements about the long-term efficacy and the recommended duration of treatment.

#### 3.1.2 Paroxetine

Kim et al. (2002) conducted an 8-week double-blind, placebo-controlled trial with 45 participants receiving either placebo or paroxetine (mean dose 51.7 mg/day) (Kim et al., 2002). Paroxetine was well tolerated, with only one dropout due to side effects. Significant improvements on the Clinical Global Impression Scale (CGI) and lower scores on the Gambling Symptom Assessment Scale (G-SAS) were observed at weeks 6–8 in the paroxetine group. Furthermore, there was a significantly higher number of responders in the paroxetine group at week 7 and 8. Comorbid psychiatric disorders were excluded and participants had low baseline depression and anxiety scores, enhancing internal validity but limiting generalizability.

A subsequent multicenter randomized placebo-controlled trial by Grant et al. (2003) included 76 participants and extended the treatment duration to 16 weeks (Grant et al., 2003). The study was similarly conducted regarding number of completers, outcome measurement instruments, methodological design and statistical tests and models used. Although improvements were greater in the paroxetine group, only the PG-YBOCS urge/thought subscale reached statistical significance (p = 0.037). In contrast to the earlier study by Kim et al., the high placebo response rate did not appear to decrease as much over time. Differences in placebo responder screening methods and randomized gender distribution in the placebo groups may have influenced the different outcomes between these two studies. While the paroxetine groups had an almost equal proportion of women, the placebo group of Kim et al. consisted of 77.3% women, but the placebo group of Grant et al. consisted of only 25% women. Even though no significant relation of gender to the outcomes was found in the final model created by using hierarchical logistic regression by Grant et al., researchers recently concluded that men on average are more susceptible to placebo effect than women and that this is induced more by verbal information than by conditioning in men (Enck and Klosterhalfen, 2019a; Vambheim and Flaten, 2017).

Grant et al. argued that unwanted behaviors in impulse control disorders may tend to decrease if more attention is paid to them. The participants’ regular clinical contacts combined with the regular completion of self-assessments may have led to improved self-awareness of their own behavior, which in turn may have had a therapeutic effect somewhat similar to cognitive behavioral therapy (CBT). They also pointed out that participants in a study are likely to show relatively high motivation to change their behavior and that a strong therapeutic relationship between participant and investigator can have an impact on treatment.

Although this second study failed to replicate the previous results, it did show a significant superiority of paroxetine over placebo in reducing unwanted thoughts and urges of gambling measured through the PG-YBOCS urge/thought score, a subsection, that Hollander et al. recommended to be used as the main primary outcome measure for future trials (Hollander et al., 2005a). Unfortunately to our knowledge there are no further studies examining the efficacy of paroxetine in GD as of today. Based on the limited evidence, no clear recommendation can be made for or against treatment with paroxetine in individuals with GD without relevant comorbidities. However, there are further hints that individuals with GD, who suffer mainly from obsessive thoughts and urges rather than impaired impulse control, may benefit from paroxetine or possibly SSRIs in general.

#### 3.1.3 Sertraline

In 2005, Saiz-Ruiz et al. conducted a 6-month double-blind, randomized controlled trial evaluating the efficacy of sertraline in the treatment of GD (Saiz-Ruiz et al., 2005). The study involved 66 participants, with a completion rate of 56%. Response was assessed using several instruments, including the Criteria for Control of PG Questionnaire (CCPGQ), PG-CGI and the South Oaks Gambling Screen (SOGS) among others. At endpoint, at an average dose of 95 mg/day, 74% of the sertraline group and 72% of the placebo group were classified as responders, with no significant differences between the two groups for neither the primary nor secondary endpoints. While these results clearly speak against the superiority of sertraline over placebo in the treatment of GD, there are a few points worth considering. For instance, the CCPGQ, which was used to determine the primary outcome (responder status), is rather atypical and may be unsuitable for this task, as it only consists of a few dichotomous questions and a more in-depth recording including improvements of gambling behavior is not possible. Since we know from previous studies that a large proportion of test subjects experience an improvement due to the placebo effect anyway, it may be even more important to identify differences in specific gambling behavior improvements. The difference between the two groups in the SOGS, a score that measures gambling behavior in a more in-depth and differentiated way, was almost significant (p = 0.06), so it is possible that a higher sample size might have yielded significant results. Nonetheless, in view of all other secondary outcomes, which did not reach anywhere near significance, it must be assumed that sertraline was not more effective than placebo in this patient group after all. As in many of the other studies, patients with comorbid depression were excluded. The authors suggested, as did many of the other researchers in this field, that subgroups that might respond better to SSRIs need to be identified. They pointed out that while there are already several studies on various differences (including gender, comorbidities, preferred type of gambling activity) in the overall population of individuals with GD, longitudinal studies are urgently needed to test the validity of these subgroups in terms of disease progression and treatment response rates (Saiz-Ruiz et al., 2005). Grant and Kim (n = 131) reported substantial lifetime psychiatric comorbidities among individuals with GD, including major depressive disorder (29%), substance abuse (35%), and impulse control disorders (18%), indicating a need for targeted treatments based on comorbidity profiles (Grant and Kim, 2001). In an older study from 1991 by Lesieur and Blume, 72 individuals with GD with a history of alcohol or drug abuse were followed-up after a combined treatment program, with improvement noted in all three areas (Lesieur and Blume, 1991). This study supports the idea that a combined therapy targeting both conditions, regardless of whether the patient is being treated primarily for GD or for their addiction, may have positive effects on both levels. Such findings could potentially be transferable to other psychiatric comorbidities and imply that early and correct diagnosis of comorbidities is essential for optimal treatment of GD.

The lack of evidence for sertraline led Saiz-Ruiz et al. to conclude that GD might bear more resemblance to an addictive disorder rather than to the notion of it being an obsessive-compulsive spectrum disorder. Interestingly enough, in the above-mentioned study by Grant and Kim, not a single one of the 131 individuals with GD examined had a past or present history of OCD (Grant and Kim, 2001). The suggestion that GD should be seen as an OCD was put forward by many early on, as there is said to be a great deal of comorbidity between the two (Grant and Kim, 2001; Blaszczynski, 1999). However, more recent studies have concluded that although individuals with GD exhibit many obsessive-compulsive features, there is only a weak link between GD and OCD and the two conditions are unlikely to be closely related (Black et al., 2010; Durdle et al., 2008). This could also explain the contradictory results on the efficacy of SSRIs in GD, even though they have been shown to be effective in the treatment of OCD (Soomro et al., 2008).

On a side note, Saiz-Ruiz et al. found that a shorter timeframe from diagnosis to treatment was significantly associated with better outcome (p = 0.004), suggesting that interventions which can facilitate early diagnosis and motivate patients to seek treatment may be highly beneficial to the GD population in general (Saiz-Ruiz et al., 2005). After all, this study provides no conclusive evidence supporting the efficacy of sertraline over placebo in the treatment of GD. The results support the reclassification of GD closer to addictive disorders rather than obsessive-compulsive spectrum disorders and emphasize the need for subgroup-specific research and early intervention strategies.

#### 3.1.4 Citalopram

We found only one open-label study of citalopram, in which 15 subjects received a mean final dose of 35 mg/d for up to 12 weeks. Thirteen patients reported improvements in urges to gamble, total number of days gambled, preoccupation with gambling and money lost on gambling (Zimmerman et al., 2002). These benefits appeared to be independent of the antidepressant effect although there were also improvements in depression and quality of life. Conversely, a case report from 2013 pointed to a possible side effect in which a patient developed a gambling addiction while taking citalopram (Cuomo et al., 2014). Although the patient’s depressive and anxious symptoms improved while taking citalopram, she began gambling, which in turn had significant financial consequences. Interestingly, there were no episodes of gambling in the past, and no gambling occurred while taking various other antidepressants. In addition, gambling behavior improved following a gradual reduction in the dose of citalopram. The exact mechanism by which citalopram may induce gambling behavior is still unclear, but animal studies have shown that certain doses increase reward sensitivity in rats (Bari et al., 2010). Even though an initial open study has shown promising results, the potential of citalopram to induce gambling should be considered.

### 3.2 Bupropion

Bupropion is a noradrenaline and dopamine reuptake inhibitor with a similar chemical structure to amphetamines. The FDA approved it as an antidepressant, seasonal affective disorder and smoking cessation drug but off-label use also includes attention deficit hyperactivity disorder (ADHD) among others (Huecker et al., 2017). Rugle et al. reported that individuals with GD often exhibited behaviors similar to ADHD during their childhood and scored significantly lower on neuropsychological assessments of attention than controls (Rugle and Melamed, 1993). According to Specker et al., there is a strong correlation between GD and impulse control disorders such as compulsive buying disorder, compulsive sexual behavior and ADHD (Specker et al., 1995). In their study, ADHD was diagnosed in 20% of the individuals with GD surveyed, while a further 18% exhibited subthreshold symptoms. With these findings, Black et al. conducted an open-label study of bupropion since studies at the time suggested its efficacy in treating ADHD in adults (Wilens et al., 2001). With a sustained-release dose of up to 300 mg/day, significant improvements in PG-YBOCS, PG-GCI, ADHD checklist and almost all other outcome measures were observed at endpoint after 8 weeks in all ten participants (Black, 2004). Due to the known high placebo response rate, these results are not very meaningful without a comparison group. However, it is worth noting that only one participant met the full diagnostic criteria for ADHD, but the mean baseline score showed mild to severe ADHD symptoms. After this short trial, Black et al. hypothesized that a subgroup of individuals with GD with co-diagnosed ADHD could benefit from specific treatment.

After the first promising studies on naltrexone, Dannon et al. published a preliminary blind-rater study in 2005 in which they compared Bupropion (up to 450 mg/day) with naltrexone (100 mg/day) in 36 individuals with GD (Dannon et al., 2005b). The study reported similar responder rates (approximately 75%) across both treatment arms, although the onset of efficacy was delayed in the bupropion group and required increasing the dosage from 300 mg/day to its maximum of 450 mg/day to achieve therapeutic benefit. These findings raised the possibility of a true pharmacologic effect of bupropion at elevated doses, even though placebo effect likely influenced outcomes.

Subsequently, a more methodologically rigorous double-blind, placebo-controlled trial by Black et al. (2007) found no statistically significant difference between bupropion and placebo in reducing GD severity as measured by the PG-YBOCS, CGI, and G-SAS (Black et al., 2007). High dropout rates (44%), small sample size (n = 39), and potential therapeutic effects of the structured assessment interviews themselves were noted as key limitations. As mentioned above, such interviews and detailed questions about gambling behavior, conducted at baseline for inclusion/exclusion and regularly thereafter to determine outcome measures, likely have a relevant therapeutic effect and contribute to the high response rate to placebos. Future studies should examine ways to reduce this effect, even if this may not be possible. The mean dose of bupropion at endpoint (324 mg/day) fell short of the maximum dose used by Dannon et al. (450 mg/day), which may have limited the observed efficacy. However, a dose of 450 mg/day is considered to be very high and should not be exceeded, as otherwise the risk of seizures and other adverse events increases significantly (Settle, 1998). In addition, bupropion should only be used with caution in patients with alcohol use disorder and/or with liver dysfunction (Huecker et al., 2025; Bupropion, 2012). Considering that alcohol use disorder and GD are highly correlated (Grant et al., 2002), this makes bupropion in high doses *per se* a rather unsuitable medication for a large portion of individuals with GD.

To date, there is neither sufficient evidence to support the use of bupropion in the treatment of GD in general, nor is there convincing evidence for its use in a subgroup with co-occurring ADHD. It is important to point out that at the time most of these studies were conducted there was good reason to believe that bupropion could be effective in improving ADHD symptoms and thus consider it a quasi-ADHD medication in trials. However, current evidence-based guidelines do not recommend bupropion for first-line treatment of ADHD but restrict its use to cases where other medications are not tolerated (Kooij et al., 2019). The strong link between ADHD and GD appears to be real (Waluk et al., 2016; Theule et al., 2019), which in turn raises the question of how individuals with GD or especially the specific subgroup with concurrent ADHD would respond to treatment with a drug approved for first-line therapy in ADHD (e.g. methylphenidate, lisdexamphetamine) (Kooij et al., 2019). To our knowledge, there are no placebo-controlled studies investigating these drugs in such a manner to date. Researchers found that psychostimulants such as amphetamine can prime motivation to gamble in individuals with GD which might be part of the reason why this angle was never examined further (Zack and Poulos, 2004). The question of how medications approved for ADHD affect gambling behavior in ADHD patients deserves further attention even if they are unlikely to be effective therapeutics for the majority of individuals with GD.

### 3.3 Nefazodone

Nefazodone belongs to the group of antidepressants, but it has a unique mechanism of action working primarily through 5-HT2A receptor antagonism as well as serotonin and norepinephrine reuptake inhibition (Davis et al., 1997). Pallanti et al. evaluated its efficacy in treating GD in an open-label study published in 2002 (Pallanti et al., 2002a). 14 outpatients without bipolar disorder (BD), schizophrenia or other psychotic disorders were assessed repeatedly over an 8-week period. Although Nefazodone led to a significant improvement in gambling behaviors measured by PG-YOBCS and PG-CGI-I, no further conclusions about efficacy can be drawn from this study alone. Due to its unfavorable safety profile regarding hepatotoxicity (Nefazodone, 2012), we suggest that limited resources for future studies be directed towards more promising drugs.

### 3.4 Opioid antagonists

#### 3.4.1 Naltrexone

Naltrexone is a long-acting opioid antagonist which is frequently used for treating alcohol and opioid addiction through competitive blockade of opioid receptors (Sudak and in, 2016). Its pharmacological action extends to modulation of the mesolimbic dopamine circuitry, particularly within the ventral striatum and nucleus accumbens which are key regions implicated in reward processing and addictive behaviors (Kim et al., 2001; Herridge and Gold, 1988; Grant et al., 2008a). By attenuating dopaminergic signaling associated with reward anticipation and reinforcement, naltrexone is hypothesized to reduce craving and compulsive behaviors not only in substance-related disorders but also in behavioral addictions such as GD.

As mentioned earlier, GD is now classified as a substance-related and addictive disorder in the DSM-V and in ICD 11. GD exhibits core characteristics of addiction (e.g., craving, compulsive engagement despite harmful consequences, tolerance, withdrawal-like symptoms) and similar brain regions to those affected by drug addiction, such as the ventromedial prefrontal cortex and striatum, are dysfunctional (Potenza, 2008; Potenza, 2013). Both shared vulnerability and mutual reinforcement effects play an important role in these disorders. Twin studies found that up to two thirds of GD and AUD co-occurrence was due to a shared genetic vulnerability, particularly among men (Slutske et al., 2013). Interestingly, the National Comorbidity Survey Replication found that while GD preceded comorbid disorders in 23.5% (whereas it followed in 74.3%), for SUDs specifically, GD more often predicted the subsequent onset of SUDs than *vice versa* (Hodgins et al., 2011). Both GD and SUDs are characterized by dysregulation of the mesolimbic dopamine reward system, though with some key differences: SUD is associated with reduced baseline dopamine D2 receptor binding and diminished reward response to drugs, whereas GD is not (Linnet, 2020). However, GD is characterized by amplified dopamine release during anticipation, a mechanism that is also highly relevant and present in drug addiction (Volkow et al., 2016). This anticipatory dopamine response may constitute a common neurobiological pathway underlying both GD and SUD, consistent with incentive-sensitization models of addiction (Linnet, 2020). The phenomenon known as cross-sensitization, whereby exposure to stress, drugs or gambling can enhance dopaminergic response to any of those stimuli, may play a central role in the development of SUD among individuals with GD (Hellberg et al., 2019).

Drug addiction involves heightened salience to drug cues, while other non-drug-related rewards are neglected, and impaired inhibitory control, particularly regarding drug use (Ceceli et al., 2025). A functional MRI study found that gambling-related stimuli are also more salient for problem gamblers and that problem gamblers rely more heavily on compensatory brain activity during response inhibition than healthy controls (Van Holst et al., 2012). These associations between cue-reactivity combined with impaired inhibitory control ultimately lead to the development of habitual behaviors in addictions (Brand et al., 2019).

Individuals with GD are much more likely to have an AUD or other SUD and *vice versa*, with estimates ranging. After analyzing 11 prevalence survey studies, a more recent systematic review and meta-analysis concluded that SUDs have a mean prevalence of 57.5% among pathological and problem gamblers (Lorains et al., 2011). Stimulant use (cocaine, amphetamine, metamphetamine) shows the strongest association with gambling behavior and can prime motivation to gamble (Zack and Poulos, 2004; Kresovich et al., 2025). Available data also suggests that alcohol consumption directly contributes to impaired self-control over gambling behavior, increased bet sizes after losses and increased gambling frequency (Baron and Dickerson, 1999; Tobias-Webb et al., 2020; Huggett et al., 2019). In particular, binge drinking patterns (at least once a week) combined with alcohol consumption while gambling increases the likelihood of risky gambling beyond either behavior alone (Smit et al., 2023). Risk consumption or alcohol dependence was also found to be a significant predictor of poor treatment outcome among individuals with GD receiving CBT (Jiménez-Murcia et al., 2016). All points considered and given their proposed mechanisms of action in addiction, this makes opioid antagonists one of the most promising class of drugs that could be used for treatment of GD.

Following an initial promising 6-week open-label trial, Kim et al. conducted a double-blind comparison study of naltrexone and placebo, which included 83 individuals with GD (Kim et al., 2001; Kim and Grant, 2001). After a one-week placebo lead-in phase, participants received naltrexone at doses titrated up to 250 mg/day over an 11-week period, with a mean endpoint dose of 188 mg/day. At the end of the study, a large proportion of the naltrexone group showed significant improvements in all outcome measures (patient- and clinician-rated PG-GCI, G-SAS) compared to the placebo group. Notably, 55% classified as “very much improved” and 20% as “much improved” with naltrexone (12% and 12% comparatively). Although 38 participants dropped out of the study, only two cases were attributed to adverse effects. A substantial portion of early terminations (n = 22) resulted from participants being classified as placebo responders based on a ≥50% reduction in G-SAS scores during the lead-in phase, highlighting the significant placebo effect commonly observed in GD trials. Elevated liver enzymes were reported in four participants, primarily associated with concurrent use of NSAIDs, but normalized upon discontinuation of naltrexone. In addition, Kim et al. found that naltrexone worked better in patients who had more pronounced urge symptoms at baseline visits compared to those with less pronounced urge symptoms, possibly making gambling urges a predictor to responsivity. Higher baseline urges and cravings are predictors of better response to naltrexone treatment in AUD (Garbutt et al., 2016). Craving is also increasingly thought to play an important role in GD as it has been described as a predictor of gambling severity and frequency, among other things (Mallorquí-Bagué et al., 2023).

Subsequent research by Grant et al. (2008) sought to replicate and expand upon these findings in a larger, 17-week RCT involving 77 subjects (Grant et al., 2008a). After a similar placebo lead-in phase, participants were randomized to receive naltrexone at fixed doses of 50, 100, or 150 mg/day. As no significant dose-response relationship was identified, data were pooled across treatment arms. Subjects receiving naltrexone had a significantly greater reduction in the PG-YBOCS total score (p = 0.0094) as well as in the PG-YBOCS subscales of urges/thoughts (p = 0.0053) and behavior (p = 0.0134). Superiority of Naltrexone was also observed across secondary outcomes, including the G-SAS and CGI. Behavioral improvements were apparent as early as 3 weeks into treatment, while significant therapeutic effect was noted by week six. Naltrexone was well tolerated, with only 6.5% of participants discontinuing due to adverse events. Overall, the results of this study clearly support the safety and effectiveness of naltrexone in the treatment of GD. One point worth noting, however, is that Grant et al. following the hypothesis by Kim et al. that gambling urge is a predictor of response to naltrexone, intended to include only subjects with pronounced urges and cravings prior to gambling behavior. To be included, patients were required to have at least a moderate craving for gambling (=2 or higher on item 1 of the G-SAS) in addition to other criteria. Unfortunately, the authors did not publish data on how many patients were excluded after screening because they scored too low on item 1 of the G-SAS, but we can assume that this was the case for at least some, which makes the study somewhat less representative overall.

Although this article is primarily concerned with studies on drug therapies and not those that actively included behavioral therapies, we felt it was important to mention the following study so as not to give the false impression that there were no conflicting results on naltrexone. Toneatto et al. conducted a randomized, double-blind study on the efficacy of naltrexone in 52 individuals with GD with concurrent AUD (Toneatto et al., 2009). 52 participants received either naltrexone (mean dose 100 ± 59.4 mg/day) or placebo over 11 weeks, along with up to seven sessions of CBT targeting both disorders. Follow-up assessments were conducted at 3-, 6-, and 12-months post-treatment. Although both groups showed substantial and sustained improvements in gambling frequency and alcohol consumption, no significant differences emerged between naltrexone and placebo.

In another study published in 2015 by Kovanen et al., 106 participants received either as-needed naltrexone (50 mg) or placebo in addition to psychosocial support over a period of 20 weeks (Kovanen et al., 2016). 68% of participants completed the entire study period, and naltrexone was used on an average of 67% of days. However, no statistically significant differences in gambling outcomes between naltrexone and placebo were observed at endpoint. Interestingly, emotional wellbeing improved significantly in a subpopulation with the AA genotype of the opioid receptor mu 1 (OPRM1) A118G polymorphism (Kovanen et al., 2016). Treatment response to naltrexone in AUD was significantly higher in carriers of the AA phenotype in a previously conducted trial by O’Malley et al. (2008), but the role of this genetic variant remains unclear since many other studies reported contradictory results (Kovanen et al., 2016).

As we have already seen, contradictory study results are not uncommon when it comes to drug treatment of behavioral disorders. While many randomized placebo-controlled trials as well as reviews and meta-analyses support Naltrexone’s efficacy in AUD (e.g. (Carmen et al., 2004; Pettinati et al., 2006; Maisel et al., 2013)), other studies also found inconclusive or contradictory results (Guardia et al., 2002; Krystal et al., 2001). A key methodological difference in Toneatto et al. study was the use of a self-monitored behavioral diary as the primary outcome measure, rather than standardized instruments such as the PG-YBOCS or G-SAS. While the authors argued that these scales may over-rely on clinician judgment and insufficiently capture behavioral change, the use of non-validated instruments limits comparability to other studies and may underestimate latent craving or cognitive preoccupations with gambling. Hollander et al. consider it problematic and do not recommend measuring only the target behavior, since in their experience individuals with GD due to their habits oftentimes go bankrupt and therefore score low on the behavior sections, when in reality they still have strong thoughts and urges to gamble (Hollander et al., 2005a). Moreover, the therapeutic impact of CBT and in-person visits in both Toneatto et al. and Kovanen et al.’s studies likely contributed to improvements in both groups, potentially masking any pharmacological effects.

Another possible explanation for Toneatto et al.’s. negative findings regarding the efficacy of naltrexone could be that AUD is associated with a generally higher severity of gambling disorder and, as previously mentioned, is a predictor for poorer treatment outcome (Lister et al., 2015). A longer treatment duration of over 11 weeks possibly may have yielded significant differences between naltrexone and placebo, as naltrexone trials on AUD that extended beyond 3 months showed greater efficacy (Murphy IV et al., 2022). However, this is speculative, and long-term treatment studies would be needed to confirm or refute this theory. Preliminary evidence also comes from a case series involving 14 patients from the UK in whom psychological interventions failed. They found that treatment with naltrexone 50 mg/day resulted in significant decreases in gambling cravings, with 60% achieving complete abstinence and a further 20% almost completely ceasing their gambling behavior (Ward et al., 2018). Two patients with comorbid AUD relapsed during naltrexone treatment, highlighting the complex intertwinement of the two disorders and their shared neurobiological mechanisms which can complicate treatment. Current guidelines recommend integrated instead of sequential treatment for AUD and co-occurring mental disorders (João and De Aquino, 2025), but no data are available specifically regarding the pharmacological treatment of GD.

In conclusion, naltrexone remains one of the more extensively studied pharmacological agents for GD. While results are encouraging, particularly for individuals with high gambling cravings and no major comorbidities, further large-scale, placebo-controlled trials are needed. Such studies should aim for methodological consistency, employ validated outcome measures, and consider stratified designs to evaluate efficacy in clinically relevant subpopulations. The relationship between baseline craving and the efficacy of naltrexone in GD warrants further investigation. Before initiating pharmacological treatment, clinicians could use screening tools to assess baseline craving levels, thereby selecting patients who potentially benefit more of naltrexone treatment.

#### 3.4.2 Nalmefene

Nalmefene is a long-acting opioid receptor modulator that is structurally closely related to naltrexone, but has a higher bioavailability and a higher affinity for central opioid receptors (Dixon et al., 1987; Soyka et al., 2016). Like naltrexone, it is an antagonist at the mu- and delta-opioid receptors, but in contrast is a partial agonist at the kappa-opioid receptor (Bart et al., 2005). Preclinical data suggest that dysregulation of endogenous opioid systems including DYN/kappa receptors likely plays an important role in alcohol dependence and possibly other addictions as well (Nealey et al., 2011). By binding and activating kappa-opioid receptors, nalmefene may reduce dopamine in brain regions that are important for the persistence of alcohol and cocaine dependence (Bart et al., 2005). Many clinical trials have so far demonstrated its efficacy in reducing heavy drinking and total alcohol consumption (Mann et al., 2013; Gual et al., 2013). A further advantage over other opioid receptor antagonists is that nalmefene does not appear to be hepatotoxic (National Institute of Diabetes and Digestive and Kidney Diseases, 2012).

In a 16-week multicenter, randomized, double-blind trial by Grant et al. involving 207 participants, nalmefene (25 mg/day, 50 mg/day and 100 mg/day) was compared to placebo (Grant et al., 2006). The 25 mg/day and 50 mg/day doses were shown to be significantly more effective than placebo in reducing gambling symptoms, as measured by the primary outcome PG-YBOCS and other secondary outcomes (e.g., CGI, G-SAS). The 25 mg/day dose yielded the most favorable balance between efficacy and tolerability. High dropout rates, particularly at higher doses, were observed, with adverse events such as nausea, insomnia, and dizziness being most common during the initial treatment phase. The authors suggested that the high initial dosing of 25 mg/day and lack of flexible titration may have contributed to these discontinuation rates.

To address these tolerability concerns, a second multi-center, placebo-controlled study (n = 233) by Grant et al. implemented a slower dose escalation strategy, starting at 5 mg/day and increasing to either 20 mg/day on week two or 40 mg/day on week three (Grant et al., 2010a). Conducted over a notably shorter treatment duration of only 3 weeks, this study found no statistically significant differences between nalmefene and placebo. However, a post-hoc analysis of those who adhered to the full target dose for at least 1 week found that 40 mg/day nalmefene resulted in significant improvements in gambling severity measured through PG-YBOCS (p = 0.035). It seems that in this study the higher dose had a better effect, but it remains unclear why it was conducted over such a short period of time, as this severely limits the validity of the results.

In a secondary analysis, Grant et al. explored predictors of treatment response to opioid antagonists using existing data from two of their previously discussed trials (Grant et al., 2008a; Grant et al., 2006). Multivariate analyses showed that neither the type of gambling nor concurrent depression or anxiety disorders were associated with respond to opioid antagonists. The strongest predictor by far was found to be a family history of AUD (p = 0.006) (Grant et al., 2008b). The authors pointed out that this finding indicates the existence of genetic predispositions which determine how a person will respond to an opioid antagonist. Such a gene variant that affects responsiveness to naltrexone in alcohol-dependent individuals has already been identified (Ray and Hutchison, 2007). Patients with concomitant AUD were excluded from both studies whose data were used by Grant et al. However, as a family history of AUD was clearly associated with a better response to an opioid antagonist, it can be hypothesized that an existing AUD may also likely be a strong predictor of response. Even though the results of the above-mentioned study by Toneatto et al. contradict this thesis, it should nevertheless be investigated in greater depth in further studies. Another finding by Grant et al. was that although baseline urges to gamble did not predict treatment response for the entire sample, they tended to be associated with higher doses, meaning that patients with strong urges responded better to high doses of naltrexone or nalmefene, which again aligns well with the results of Kim et al. study on naltrexone (Kim et al., 2001).

Overall, there are unfortunately not enough studies on the effect of opioid antagonists in GD and the existing ones are partly contradictory. Many questions remain unanswered, such as the optimal dosage, the long-term effect beyond the acute phase or which patients are more likely to benefit from different opiate antagonist. However, the study by Grant et al. suggests that available clinical information such as a family history of AUD can be used to select distinct patient groups for more specific pharmacotherapy. Furthermore, it can be said that in the case of individuals with GD who are at high risk of liver damage or who already have such damage, nalmefene should rather be administered to these patients due to the dose-dependent liver toxicity of naltrexone. Despite the small number of studies, a recent systematic review and network meta-analysis concluded that naltrexone and nalmefene are currently the most evidence-based drug therapy for treatment of GD (Ioannidis et al., 2025).

#### 3.4.3 Naloxone

Unlike naltrexone and nalmefene, naloxone is a short-acting competitive opioid antagonist with the highest affinity for the mu-opioid receptor, while also binding to the kappa- and delta-opioid receptors in the central nervous system (Jordan et al., 2025). Accordingly, it is mainly used as an antidote for opioid overdose with respiratory insufficiency.

Based on some of the promising results of the studies discussed above, naloxone’s efficacy in the treatment of GD was also investigated. In 2021, Alho et al. published the results of their double-blind RCT comparing intranasally administered naloxone with placebo. Although gambling symptoms improved, naloxone did not prove superior to placebo (Alho et al., 2022). This trial had a relatively high number of completers (n = 106) and relatively broad inclusion criteria, particularly with regard to concurrent alcohol consumption. A key difference from other studies on opioid antagonists is that in those, the drug was administered on a fixed schedule, whereas here it was administered as-needed. Only 28% of participants used the drug correctly throughout the study period according to the preset compliance criteria (Alho et al., 2022). Combined with the significantly shorter half-life of naloxone, this may have compromised its efficacy. The authors reiterated that the psychosocial support provided, the motivational interventions, and the use of self-help materials may have been so effective that they masked any differences between naloxone and placebo. Furthermore, the 12-week study period may have been too short to achieve statistically significant differences, as opioid antagonists administered as-needed have shown benefits in AUD even after a longer period of time (Mann et al., 2013). The authors concluded that IN naloxone may be better tolerated than orally administered opioid antagonists and should therefore be further investigated for the treatment of GD.

### 3.5 Lithium

Lithium is a monovalent cation with strong mood-stabilizing and anti-suicidal effects, making it an effective treatment for acute manic episodes and for long-term phase prophylaxis in BD (Keck and McElroy, 2002). Among other indications are episodes of unipolar depression and schizoaffective disorder. Its pharmacodynamics are not fully understood yet, but its therapeutic effects likely stem from reduced excitatory (primarily dopamine and norepinephrine) neurotransmission and interfering with intracellular neuronal second messenger signaling pathways through enzyme inhibition and alteration (Brown and Tracy, 2013). Furthermore it shows neuroprotective abilities and could be beneficial in prevention of dementia (Barroilhet and Ghaemi, 2020). Multiple studies suggest that GD and BD are linked to each other and oftentimes co-occur. Among common clinical features are impulsive behavior without reflection, affective symptoms like mood swings and overestimating one’s own abilities (McElroy et al., 1996; Pallanti et al., 2002b). If the urge to gamble is not satisfied, individuals with GD often experience agitation, restlessness, tension and/or anxiety, which resemble both elevated and depressive mood states in BD (Cunningham‐Williams et al., 2009; Di Nicola et al., 2014). Like mania/hypomania, increased need for pleasure in combination with lack of control over impulsivity result in harmful and reckless behaviors for individuals with GD (McElroy et al., 1996; Di Nicola et al., 2014). McIntyre et al. found that the prevalence of GD is significantly higher among patients with bipolar I disorder than in patients with major depression or in general population (McIntyre et al., 2007). Individuals with BD are estimated to be up to four times more likely to develop problem gambling compared to those without BD (McIntyre et al., 2007; Jones et al., 2015).

In view of these strong associations between the two disorders and various promising case reports, Hollander et al. conducted a double-blind, randomized and placebo-controlled study on the efficacy of sustained-release lithium in individuals with GD who were also diagnosed with bipolar spectrum disorder (bipolar II, BD not otherwise specified, or cyclothymia) (Hollander et al., 2005b). The study explicitly excluded individuals with bipolar I disorder, likely due to ethical concerns surrounding placebo use in this population. A total of 40 subjects were randomly assigned to receive either sustained-release lithium or placebo for a period of 10 weeks. Lithium appeared to be clearly superior to placebo: the lithium group had a significantly lower PG-YBOCS score (p < 0.001) and had greater improvements on the PG-CGI score (p = 0.001) at endpoint, results that were confirmed by multiple ITT analyses. Findings on respective responder status were equally impressive with 83% of the lithium group and only 29% of the placebo group considered responders. In addition, but perhaps not surprisingly, the subjects who received lithium also showed significantly fewer manic symptoms and impulsivity. The mean dose of lithium at endpoint was 1150 mg with mean level 0.87 mEq/L but a higher blood level did not result in significantly greater improvements.

Although these results are not transferable to the general population, they demonstrate how effective targeted medication can be for a specific subgroup among individuals with GD and how important it is to diagnose specific comorbidities correctly and at an early stage. The authors suggested that individuals with GD should be subtyped and treated according to their comorbid symptom profile, highlighted by the fact that an injudiciously given SSRI, for example can exacerbate hypomanic symptoms or even induce full-blown mania (Hollander et al., 1998; Breggin, 2004). Of note, over 45% of all subjects who applied for this study were classified as suffering from a bipolar spectrum disorder based on the “Mood Disorder Questionnaire”. Even if one factors in that these subjects were not necessarily representative of the total number of individuals with GD and that patients with bipolar symptoms may have been disproportionally invited to enrollment, this is still an astonishingly high number which further hints to the intertwinement of these two disorders. Despite the clear results of this study, the main limitation of the relatively short observation period must be considered. From all the previously discussed trials it is now obvious that the vast majority of placebo-controlled studies on GD are conducted over a short period of time and that there is very few research on the long-term efficacy of the various drugs. While lithium appears to be effective in the treatment of GD in an initial acute phase, the effect on gambling behavior in the longer term remains unclear. In most cases, long-term treatment with a blood level of at least 0.8 mEq/L is indicated for phase prophylaxis of BD (Chokhawala et al., 2025). If patients with comorbid GD are willing to take lithium long-term, one can hypothesize it will also have a positive effect on gambling behavior if it is indeed effective beyond an initial phase. To confirm this, long-term observational studies investigating both bipolar and GD symptoms are needed. Furthermore, this trial showed a significant correlation between improvements in gambling behavior and mood stability, but the question of causality and the exact association of the two remains open. Both that increased affective stability induced by lithium leading to less gambling or that lithium specifically reducing impulsive gambling and thus increasing affective stability, are conceivable. Since the improvements in gambling scores were greater relative to those in the clinician-administered rating scale for mania and, in addition, no significant differences between the two groups were found in HAM-A and HAM-D scores, Hollander et al. also considered the possibility of lithium having a strong effect on the specific impulses that lead to gambling behavior. Lithium has been shown to reduce impulsive action in both animal and human studies, so this hypothesis is rather well founded (Sheard et al., 1976; Ohmura et al., 2012). Another interesting question is whether lithium would generally be the drug of first choice for individuals with GD with comorbid BD, or whether other drugs should be preferred depending on if a depressive or manic phase is prominent. This could be particularly relevant as lithium, unlike many other regularly used drugs, has a narrow therapeutic range and therefore requires high compliance as well as regular monitoring of thyroid and renal function (Chokhawala et al., 2025). To our knowledge, with one exception, there are no other placebo-controlled studies to date in which lithium or other medications approved for BD have been studied in individuals with GD with comorbid BD (topiramate not included). The one exception concerns olanzapine and the relevant trial is discussed later in this paper.

### 3.6 Valproate

Valproate is the stable, coordinated compound of sodium valproate and valproic acid (Rahman et al., 2025). Due to its strong antiepileptic properties, it has been approved by the FDA for the treatment of a variety of seizure disorders (Rahman et al., 2025). Besides its antiepileptic effects, valproate acts as a powerful mood stabilizer and is frequently used in the treatment of BD, particularly during manic episodes. The strong association between GD and BD in combination with the promising results of the study by Hollander et al. on lithium (Hollander et al., 2005b) make valproate an interesting candidate for this patient group.

In 2002, Pallanti et al. published the results of their randomized single-blind study investigating the effect of lithium and valproate on individuals with GD without relevant comorbidities (Pallanti et al., 2002b). Both drugs showed similarly good efficacy, but there was no placebo group for comparison. Additional supportive evidence, although preliminary, comes from smaller studies and case reports. For example, a small-scale study involving 13 subjects receiving valproate in combination with CBT found significant improvements in gambling behavior (Vasile et al., 2011). In a more recent case report, a Parkinson’s disease patient who had developed severe GD behavior and multiple other impulse control/behavioral disorders due to his dopaminergic medication experienced complete remission of gambling symptoms within 1 month of initiating valproate treatment (Degirmenci and Kececi, 2019). Interestingly, prior adjustments and dose reductions of his antiparkinsonian medication had mitigated other behavioral issues but had no impact on his gambling behavior.

There is anecdotal evidence for valproate in the treatment of GD, but no RCTs have been conducted to date. Of particular interest is its potential effect on patients with concurrent BD, as it has been successfully and widely used here already. In addition, several studies indicate that valproate could also be effective in the treatment of impulsive, aggressive behavior in borderline personality disorder (Kavoussi and Coccaro, 1998; Hollander et al., 2001). We suggest that future studies focus on these areas and specifically compare the efficacy of valproate with lithium and placebo. Even if valproate proves to be inferior to lithium, its relatively favorable safety profile and tolerability could still make it a viable alternative.

### 3.7 Topiramate

Topiramate is a second-generation antiepileptic drug approved by the FDA for both monotherapy and adjunctive treatment of epilepsy, as well as for migraine prophylaxis (Fariba and Saadabadi, 2025). Over recent years, it has also been increasingly used off-label in the treatment of various psychiatric disorders including behavioral addictions and SUDs (Arnone, 2005; Paino et al., 2020). Its mechanism of action in psychiatric disorders is not fully understood yet, but it appears to reduce impulsivity, cravings and urges, clinical characteristics which all play an important role in many of these disorders. Efficacy in the treatment of AUD (Guglielmo et al., 2015) as well as in eating disorders (Arbaizar et al., 2008) has been demonstrated in multiple studies. Nicolato et al. reported a case of a patient with bipolar II disorder and comorbid GD who did not respond to lithium monotherapy but experienced complete and sustained remission of gambling behavior following the addition of topiramate at 200 mg/day (Nicolato et al., 2007). The previously discussed study by Dannon et al. comparing fluvoxamine with topiramate also suggested that topiramate might be effective, but as we know there was no placebo comparison group (Dannon et al., 2005a).

Based on the promising studies and its unique pharmacological properties, Berlin et al. conducted a multicenter, double-blind, randomized, placebo-controlled study on topiramate in the treatment of GD (Berlin et al., 2013). Forty-two participants including such with subsyndromal affective symptoms were randomized to receive either topiramate (up to 300 mg/day) or placebo over 14 weeks. The primary outcome was defined as changes in PG-YBOCS obsession subscale while secondary outcomes included PG-YBOCS total and compulsion subscales, G-SAS, CGI-I, and Barratt Impulsivity Scale (BIS-11). At endpoint, 65% of participants were rated as responders but no significant differences were found between topiramate and placebo on any outcomes. Trends toward improvement in BIS-11 total and subscales were noted but did not reach statistical significance. The authors pointed out the possibility of a type II error, i.e., that smaller improvements compared to placebo may not have been detected due to the smaller-than-planned sample size. Initially, they wanted to enroll 120 subjects, but the strict exclusion criteria resulted in a slow process and eventually the funds ran out prematurely. They suggested using less stringent exclusion criteria for future studies in order to find a sufficiently large number of participants more easily and quickly and to find a “realer” patient group that is more representative of the overall population of individuals with GD. As is so often the case, the pronounced placebo effect experienced in this trial is probably also a major limiting factor. Apparently, the authors did not take any measures, such as a placebo lead-in phase, to weed out strong responders and reduce the overall placebo effect. The subjects who completed the study met with the staff at least 10 times during this time.

Based on these results, no recommendation for topiramate can be made, but we would not completely rule it out as a possible drug for the treatment of GD, if only because of the lack of research and RCTs to date. Although the trial by Berlin et al. did not demonstrate general efficacy of topiramate, it may indicate its efficacy in patients with predominantly impulsive clinical features. Since it has also been used successfully in the treatment of AUD, topiramate may be an option for individuals with GD with comorbid AUD. With the evidence available today, an opiate antagonist would likely be tried first in such a patient, but topiramate could be considered in case of contraindications or non-response. Findings from animal studies also suggest that a combined therapy of topiramate and an opiate antagonist may be more effective in the treatment of AUD than a single drug therapy (Moore C. F. et al., 2014). The question of its effect with combined lithium therapy in comorbid BD also remains open. To better target resources, future studies on topiramate could focus on individuals with GD who fall into the “impulsive subtype” and/or on those with concurrent AUD.

### 3.8 Olanzapine

Olanzapine is an atypical antipsychotic drug that exerts its action primarily as an antagonist of dopamine D2 and serotonin 5-HT2A receptors and as such is generally used for treatment of schizophrenia (Keck and McElroy, 2002). However, it is also frequently used for treatment of BD (Tohen et al., 2003; Tohen et al., 1999). Given the strong comorbidity between GD and BD (McElroy et al., 1996; Petry et al., 2005), the positive findings by Hollander et al. (Hollander et al., 2005b), and emerging research indicating that the function of the dopaminergic system is altered in individuals with GD (Bergh et al., 1997), researchers have questioned whether olanzapine may also offer therapeutic benefit in GD.

To date, two double-blind, randomized, placebo-controlled trials have evaluated the efficacy of olanzapine for GD. The first study conducted by McElroy et al., enrolled 42 participants who were randomized to receive either olanzapine or placebo over a 12-week treatment period, preceded by a one-week placebo lead-in phase and followed by a one-week discontinuation phase (McElroy et al., 2008). The primary outcome measure was PG-YBOCS while secondary outcome measures included self-report diaries in which the frequency of gambling episodes and total hours spent gambling were recorded weekly. At endpoint, both groups demonstrated improvements, but no significant differences were found between olanzapine and placebo across any outcome measures. In addition, almost twice as many patients in the olanzapine group discontinued the study prematurely compared to the placebo group (52% versus 28.6%) (McElroy et al., 2008). The tolerability of olanzapine was mediocre, with significant weight gain (p = 0.0015) being particularly problematic. This is a known side effect of atypical antipsychotics, which occurs in up to 50% of patients undergoing long-term treatment and is a major reason for discontinuation for many (Ascher-Svanum et al., 2010). Given the apparent lack of efficacy combined with the very real side effects, olanzapine appears to be a rather unsuitable medication for GD. McElroy et al. further noted that the trial was adequately powered to detect even modest treatment effects and calculated the likelihood of a type II error as very low (<3%).

One point worth considering is, as in the study by Hollander et al. (2005b), subjects with bipolar I disorder were excluded, but other bipolar disorders were allowed in this trial. However, in contrast to the prior study, only eight participants (19%) had a lifetime prevalence of soft-spectrum BD and since those were randomly assigned to both groups, the actual number of subjects with a history of BD who received olanzapine, was very low. It is therefore possible that olanzapine would have been more effective in a larger sample of patients with comorbid BD. As in many GD studies, the placebo response was high (∼70%), and the placebo lead-in phase did not appear to mitigate this effect. Again, the high response rates may result from the self-monitoring through diaries and the relatively high number of clinician visits (at least 12 visits for completers.

The second RCT was conducted by Fong et al. and investigated the effect of olanzapine on pathological video poker players. In contrast to McElroy et al., all major Axis I disorders were excluded, slightly different outcome measures were used, and medication was administered for only 7 weeks (Fong et al., 2008). However, they came to very similar results: Although both groups showed a decrease in gambling behavior over time, no statistically significant superiority of olanzapine over placebo was found. Similarly, self-reporting diaries have been used extensively in this trial with subjects recording their gambling behaviors daily.

Based on the current limited evidence, olanzapine and probably other antipsychotics as well do not appear to be effective medications for the majority of individuals with GD. However, a case report from 2001 described a woman suffering from schizophrenia and GD in whom gambling behavior ceased after regular intake of 10 mg olanzapine (Potenza and Chambers, 2001). Since GD is more common in individuals suffering from schizophrenia and schizoaffective disorder (Desai and Potenza, 2009), future studies should target these select subgroups where dopaminergic dysfunction is more present and where antipsychotics are already standard care. Particular caution is warranted when prescribing antipsychotics which act as partial dopamine agonists (e.g., aripiprazole, brexpiprazole, cariprazine) as they are associated with an increased risk of GD and other impulse control disorders (Moore T. J. et al., 2014). Dopamine agonists can induce GD by overstimulating D3 receptors in the ventral striatum (Moore T. J. et al., 2014). This can lead to impaired cognitive abilities relevant to impulse control, particularly impairing the brain’s ability to process losses while simultaneously amplifying the salience of winning experiences (Mortazavi et al., 2023). Partial dopamine agonists should therefore, if possible, not be used in patients who already exhibit problematic gambling behavior.

### 3.9 N-Acetyl Cysteine

N-Acetyl Cysteine (NAC) is an active ingredient with mucolytic and antioxidant properties which is mainly used to loosen mucus in chronic bronchial diseases or as an antidote to acetaminophen poisoning (Ershad et al., 2025). Animal studies have shown that glutamate levels within the nucleus accumbens play a critical role in reinstatement of drug-seeking behavior in cocaine-dependent laboratory rats (McFarland et al., 2003). Repeated cocaine use appears to induce brain plasticity leading to decreased cystine-glutamate exchange and increased excitatory signaling within corticostriatal pathways (Amen et al., 2011; Madayag et al., 2007). Pharmacological modulation of glutamate release may therefore effectively reduce cravings in cocaine dependence and possibly other addictions as well. NAC has been shown to activate cysteine-glutamate exchange, alter the plasticity-dependent effects of cocaine and reduce drug desire in cocaine-dependent patients (Amen et al., 2011; Madayag et al., 2007; LaRowe et al., 2007).

Based on these promising findings regarding cocaine addiction, Grant et al. hypothesized it could also reduce cravings in gambling and conducted the first pilot study. 27 individuals with GD were enrolled and treated with a mean effective dose of NAC 1476.9±311.3 mg/day in an open-label manner over a period of 8 weeks (Grant et al., 2007). At the end of the open-label phase, 16 subjects (59.3%) showed a ≥30% decrease in PG-YBOCS and were therefore classified as responders with 10 of 16 responders even showing a complete abstinence from gambling. 13 responders participated in the second double-blind phase and were randomly assigned to NAC or placebo for 6 weeks. At study endpoint, 83.3% of the NAC group were still considered responders, compared to only 28.6% of the placebo group. The differences between NAC and placebo were not statistically significant but approached significant levels in the PG-YBOCS total score (p = 0.095), thought/urge subscore (p = 0.06) and G-SAS (p = 0.06). It is quite possible that a larger sample size would have led to clear statistically significant differences here. Although various comorbid conditions (e.g., BD or psychotic disorders) were excluded from the study, a relatively large number of them were also included: almost a third of participants had a lifetime history of major depressive disorder and a fifth had a history of AUD. Interestingly, only one participant reported using drugs, apart from nicotine and alcohol, suggesting that NAC may indeed be effective in treating behavioral addictions beyond substance dependence.

Encouraged by their earlier findings, Grant et al. conducted another study on individuals with GD with co-occurring nicotine dependence where NAC was added to therapy with imaginal desensitization (Grant et al., 2014). During the 3-month follow-up period, NAC proved superior to placebo in reducing gambling symptoms and was also effective, at least in the short term, in reducing nicotine dependence. The authors concluded that additional NAC treatment during behavioral therapy facilitates long-term application of therapeutic techniques (Grant et al., 2014). This paper demonstrates the importance of follow-up assessments in identifying the potential effects of a drug or verifying its lasting efficacy. Unfortunately, to our knowledge, there are no other studies that have investigated the effect of NAC on GD. Future studies should repeat similar follow-up periods and should examine NAC’s efficacy, particularly in individuals with comorbid cocaine or nicotine dependence.

### 3.10 Disulfiram

Several case reports and preclinical studies suggest that disulfiram can reduce the urge to gamble in individuals with GD and co-occurring alcohol dependence (Mutschler et al., 2010a; Mutschler et al., 2010b; Müller et al., 2011; Di Ciano et al., 2018). Disulfiram is used primarily to treat alcohol dependence through deterrence because it inhibits the enzyme acetaldehyde dehydrogenase, which in turn causes very unpleasant symptoms such as nausea and dizziness when alcohol is consumed (Wright and Moore, 1990; Mutschler et al., 2009). Another mechanism of action is the inhibition of dopamine β-hydroxylase, which in turn increases dopamine levels and decreases noradrenaline levels in the brain (Müller et al., 2011; Di Ciano et al., 2018). This has already been shown to be reasonably effective in the treatment of cocaine addiction (Petrakis et al., 2000). In a double-blind randomized study published in 2017, Grant et al. examined the efficacy of disulfiram in alcohol-dependent veterans with comorbid gambling addiction (Grant et al., 2017). They found that treating alcohol addiction with disulfiram was less effective if the patients also showed problem gambling features. Unfortunately, no data on gambling behavior was collected, which is why no further conclusions can be drawn here. Overall, the promising case reports warrant further controlled studies to examine Disulfiram’s efficacy and understand the underlying mechanisms in treating GD.

### 3.11 Acamprosate

Acamprosate is an active substance currently approved by the FDA in combination with psychosocial support for maintenance of abstinence of alcohol (Center for Substance and Abuse Treatment, 2009). Various studies have shown that acamprosate prolongs the time to relapse compared to placebo (Paille et al., 1995; Sass et al., 1996), which is why, unlike opioid antagonists, it is mainly used in patients who want to remain completely abstinent. Its mechanism of action is primarily based on modulation of the glutamatergic system, specifically by interactions with N-methyl-D-aspartic acid receptors (NMDA receptors) and possibly also with metabotropic glutamate receptors (Al Qatari et al., 1998).

As mentioned earlier, glutamatergic circuits play an important role in the development of addiction, which automatically makes acamprosate a potentially promising drug in this context. However, a preliminary study examining the effects of acamprosate and baclofen on 17 individuals with GD concluded that neither substance had any effect on gambling abstinence (Dannon et al., 2011). Conversely, an open-label study from Black et al. suggested that acamprosate may be effective and well tolerated in GD. Significant improvements were observed in several measures of gambling severity and associated symptoms, with 65% of participants showing much or very much improvement (Black et al., 2011). Neither of the two studies was double-blind or randomized, which significantly limits the validity of the results. At present, the use of acamprosate for the treatment of GD is experimental at best and further studies are needed.

### 3.12 Memantine

Memantine exerts its effect mainly by modulating NDMA (N-methyl-D-aspartate) receptors which are involved in glutamate neurotransmission (Johnson and Kotermanski, 2006). In a pilot study published in 2010, Grant et al. hypothesized that memantine could reduce cravings in GD and additionally improve cognition by modulating glutamatergic neurotransmission in the prefrontal cortex (Grant et al., 2010b). Over a period of 10 weeks, participants showed a decrease in scores on the Yale PG-YBOCS from an average of 21.8 to 8.9. In addition, the same study reported improvements in cognitive flexibility, which were reflected in a significant reduction in errors in the intra-dimensional/extra-dimensional (ID/ED) set-shift task.

Anecdotal evidence comes from a case report of a 23-year-old patient who experienced a notable reduction in thoughts, tension, and excitation associated with GD after the introduction of memantine in addition to his other psychotropic medications (Pavlovic, 2011).

### 3.13 Amantadine

Amantadine is a multifunctional drug originally developed as an antiviral medication that is now primarily used in treating Parkinson’s disease due to its dopaminergic activity. Its mechanisms of action include enhancement of dopamine release, inhibition of dopamine reuptake, and antagonism of N-methyl-D-aspartate (NMDA) receptors (Bailey and Stone, 1975; Stoof et al., 1992). Amantadine also modulates glutamate transmission, which may reduce excitotoxicity and has led to investigations into its use in neuropsychiatric and addictive disorders (Nakano et al., 2019). Impulse control disorders are strongly associated to dopaminergic therapy and occur in approximately 17% of patients taking such medication (Voon et al., 2017).

In a double-blind, placebo-controlled, crossover study by Thomas et al., 17 Parkinson’s disease patients exhibiting GD received either amantadine (200 mg/day) or placebo. Amantadine proved superior over placebo, as all treated patients either completely stopped gambling or showed a significant reduction in gambling behavior measured via validated scales (Thomas et al., 2010). Interestingly, changes in gambling behavior already occurred within a few days of treatment. Intrigued by these results, Weintraub et al. analyzed data from a large cross-sectional study on impulse control disorders in Parkinson’s disease. However, they found that amantadine use was associated with a higher rate of impulse control disorders including GD (Weintraub et al., 2010).

In a case report from 2012, a 47-year-old patient reported reductions from extreme to mild-moderate gambling urges after treatment with Amantadine, suggesting potential efficacy in patients without PD (Pettorruso et al., 2012; Pettor et al., 2014). Given the limited and conflicting evidence, further large-scale studies would be needed to evaluate the efficacy and safety of amantadine. Despite the findings of Weintraub et al., we suggest that future studies focus on the role of amantadine in the treatment of GD associated with PD.

### 3.14 Gabapentinoids

Gabapentinoids primary mechanism of action involves inhibition of voltage-gated calcium channels in the central nervous system, resulting in decreased release of several excitatory neurotransmitters including glutamate and noradrenaline (Scott et al., 2003; Alles et al., 2020; Houghton et al., 2017; Reyes Fernandez et al., 2022). They are approved for the treatment of neuropathic pain and epilepsy, conditions associated with neuronal hyperexcitability (Jensen, 2002; Yasaei et al., 2025; Cross et al., 2025). Pregabalin, and to a lesser extent Gabapentin, have also demonstrated efficacy in treatment of alcohol use disorder and alcohol withdrawal (Martinotti et al., 2010; Mason et al., 2014).

In a six-month pilot trial, Pettoruso et al. investigated several medications with effects on glutamatergic transmission on GD. Both Pregabalin and Gabapentin had a significant effect on reducing gambling craving and behavior as well as improving treatment retention (Pettorruso et al., 2013). In the aforementioned case report of citalopram-induced GD, the patient was treated with pregabalin and alprazolam in addition to a gradual reduction in dosage, which eventually led to a reduction in gambling symptoms (Cuomo et al., 2014). Given the growing consensus on the key role of glutamate homeostasis and glutamate circuits in drug addiction and possibly also in behavioral addictions, the role of gabapentinoids in this regard warrants further investigation (Potenza, 2008; Kalivas, 2009; Krupitsky et al., 2007). However, they also pose a risk for misuse and abuse, particularly among individuals with a history of substance use disorders (Evoy et al., 2021).

### 3.15 Modafinil

Modafinil is a wakefulness-promoting agent used primarily to treat narcolepsy, obstructive sleep apnea, and shift work sleep disorder (Kumar, 2008). Also among its many off-label use cases is treatment of cocaine addiction (Kampman et al., 2015). Through inhibition of dopamine transporters, it increases dopamine levels in reward-related brain regions but binding differently than cocaine results in less euphoria and therefore less potential for abuse (Martínez-Raga et al., 2008; Schmitt and Reith, 2011). In addition to its dopaminergic effects, modafinil also increases extracellular glutamate levels in various regions of the brain (Minzenberg and Carter, 2008).

Zack and Poulos conducted a double-blind, placebo-controlled study to determine whether modafinil (200 mg/day) reduces the reinforcing effects of slot machine gambling in individuals with GD and whether this effect is stronger in individuals with high versus low impulsivity (n = 20) (Pettor et al., 2014; Zack and Poulos, 2009). Under Modafinil, the mean bet size decreased around 10% in both groups. Apart from that it had bi-directional effects: in high impulsivity subjects, modafinil decreased desire to gamble, salience of gambling words, disinhibition and risky decision-making while it increased scores on these indices in low impulsivity subjects (Zack and Poulos, 2009). The authors suggest that the dopamine D2 receptor plays an important role in this bidirectional response not only with modafinil but also with other D2 agonists. They hypothesized that modafinil may trigger GD in non-impulsive individuals, similar to how dopamine agonist therapy can trigger GD in Parkinson’s patients (Zack and Poulos, 2009). This is supported by several case reports in which narcolepsy patients developed GD after treatment with modafinil and the symptoms resolved after switching to other medications (George et al., 2015; Shin et al., 2023). A prospective study analyzing Zack and Poulos’ data found that modafinil may deter individuals with GD from chasing losses but also encourage them to continue betting rather than quit while they are ahead (Smart et al., 2013).

Although modafinil remains an interesting potential treatment for various conditions, current evidence does not support its universal use in GD. Special caution should be used in individuals with low impulsivity as it can be harmful in this group. Future studies could investigate the dose-dependent efficacy of modafinil in patients with comorbid ADHD or in patients with generally high impulsivity.

### 3.16 GLP-1 receptor agonists

Several emerging drugs are currently being researched for their role in the treatment of behavioral addictions. Among the most promising medications are GLP-1 receptor agonists, whose efficacy in treating AUD and other SUDs has been demonstrated consistently in multiple preclinical studies (Farokhnia and Leggio, 2026; Völker et al., 2025). GLP-1 receptors are expressed in key reward-processing brain regions including the nucleus accumbens, ventral tegmental area (VTA) and amygdala, all of which play a central role in addiction cycles (Farokhnia and Leggio, 2026). By modulating dopamine signaling and glutamatergic neurotransmission in the nucleus accumbens and the VTA, as well as by decreasing functional connectivity within the amygdala, GLP-1 agonists collectively attenuate reward processing and are hypothesized to thereby reduce addictive behavior (Egecioglu et al., 2013; Mietlicki-Baase et al., 2014; Merkel et al., 2025; Au et al., 2025). Although there are currently no published data on the specific effects of GLP-1 agonists on GD, given the similar dysfunctions between SUDs and GD in the aforementioned brain regions, one can assume that they could also represent an effective treatment option for GD.

### 3.17 Ketamine/Esketamine

Other emerging medications for the treatment of different forms of addiction include ketamine and esketamine. Various studies have shown promising results regarding AUD, cocaine addiction and opioid dependence, particularly when combined with psychological interventions (Grabski et al., 2022; Dakwar et al., 2019; Gent et al., 2024). They can modulate the glutamatergic system through NDMA-receptor antagonism, resulting in downstream effects which can rapidly enhance neuroplasticity (within hours) in the prefrontal cortex and other brain areas (Lewis et al., 2024; Hess et al., 2022; Aleksandrova and Phillips, 2021). Their exact mechanism of action in addiction treatment is unknown but may involve disruption of functional neural networks, treatment of comorbid depressive symptoms and blocking reconsolidation of drug-related memories (Ezquerra-Romano et al., 2018). A recent study examined the neurobiological mechanisms of ketamine and its impact on problem gambling: although no significant improvements were observed in gambling-related measures, ketamine significantly increased visual long-term potentiation (LTP), suggesting potential positive effects on neuroplasticity (Krecké et al., 2025). Ketamine may restore baseline reward function (impaired in depressive disorder) while simultaneously disrupting hyperactive reward circuits (Yao et al., 2018), which could make it a particularly effective treatment for GD with comorbid depressive disorder. Further studies are needed to investigate the baseline- and dose-dependent effects of ketamine and esketamine on LTP and their implications for GD.

## 4 Discussion

Despite nearly 30 years of research in clinical and controlled trials, no medication has yet emerged as the clear best treatment option for GD. Table 1 provides a summary of the results, data, our recommendation, the mechanism of action and medication group, as well as the advantages and disadvantages of the off-label medications we evaluated. Current evidence favors opioid antagonists as the primary pharmacological treatment for GD for most patients (Ioannidis et al., 2025; Dowling et al., 2022), but it is limited by the small number of respective studies and a general lack of knowledge on long-term efficacy of these agents. Research into psychotropic drugs for GD has faced several challenges: studies often show a high placebo response rate, small sample sizes, short study durations, and limited demographic diversity. In addition, subtypes of GD and potential neurobiological or pharmacogenetic markers have hardly been investigated to date.

**TABLE 1:** Off-label and investigational drugs in the treatment of pathological gambling: overview.

Medication	Results	Data	Recommendation	Mechanism of action	Medication group	Advantage	Disadvantage
SSRIs	Conflicting	Good	Ensure adequate treatment of comorbid anxiety and affective disorders to avoid poorer long-term outcome	Selective serotonin reuptake inhibitor	Antidepressants	Favorable safety profile Efficacy in multiple psychiatric disorders	Likely minor effect on gambling behavior Possible induction of gambling behavior (citalopram)
Bupropion	Disappointing (safety concerns)	Intermediate	Not useful for majority of individuals with gambling disorder	Norepinephrine and dopamine reuptake inhibitor	Antidepressant	Potential efficacy in very high doses	Adverse events (particularly in comorbid alcohol use disorder)
Nefazodone	Disappointing (safety concerns)	Poor	Not useful for treatment of gambling disorder	5-HT2A receptor antagonist Serotonine and norepinephrine reuptake inhibitor	Antidepressant	Unique mechanism of action	Hepatotoxicity
Naltrexone	Promising	Good	First-line therapy (particularly for GD with strong cravings and family history of alcohol use disorder) Further clinical testing	Long- acting mu-/kappa-/delta-receptor opioid antagonist	Opioid antagonist	Efficacy across multiple addiction types No misuse potential	Adverse events including hepatotoxicity
Nalmefene	Promising	Intermediate	First-line therapy (particularly with family history of alcohol use disorder) Further clinical testing	Long-acting mu-/delta-receptor opioid antagonist Partial kappa-receptor agonist	Opioid antagonist	Efficacy in alcohol use disorder No hepatotoxicity Greater bioavailability than Naltrexone	Adverse events Oral formulation not available in the US
Naloxone	Conflicting	Limited	Further clinical testing	Short-acting mu-/delta-/kappa- receptor opioid antagonist	Opioid antagonist	Rapid onset of action with as-needed therapeutic potential	No superiority over placebo in RCT
Lithium	Promising in comorbid bipolar disorder	Limited	Further preclinical testing and clinical testing in subpopulation with comorbid bipolar disorder	Unknown mechanism of action	Mood stabilizer	Superior efficacy for bipolar disorder Strong anti-suicidal properties	Requires high compliance and monitoring
Valproate	Promising	Poor	Further preclinical and clinical testing (particularly in subpopulation with comorbid bipolar disorder)	Inhibition of GABA transaminase	Anticonvulsant	Multiple off-label use cases Favorable safety profile	Teratogenicity Contraindicated in hepatic disease
Topiramate	Conflicting (promising)	Intermediate	Further preclinical and clinical testing	Selective antagonist of GluK1 kainate receptor Voltage-gated calcium channel inhibitor	Anticonvulsant	Promising results in treatment of alcohol use disorder Potential additional therapy	Adverse events
Olanzapine	Disappointing	Intermediate	Not useful for majority of individuals with gambling disorder	Dopamine D2-antagonist	Antipsychotic	Potentially useful in comorbid schizophrenia	Adverse events (particularly weight gain)
N-Acetyl cysteine	Promising	Intermediate	In combination with behavioral therapy (particularly in comorbid cocaine and/or nicotine dependence) Further preclinical and clinical testing	Activation of cystine-glutamate antiporter (system xc-)	Mucolytic	Excellent safety profile Low cost Broad applicability	Gastrointestinal side effects at high doses
Disulfiram	Conflicting	Poor	Further preclinical and clinical testing	Inhibition of aldehyde dehydrogenase Inhibition of dopamine-beta-hydroxylase	Medication for alcohol use disorder	Multiple promising case reports	Hepatotoxicity Not suitable for patients who want to continue alcohol consumption
Acamprosate	Conflicting	Poor	Further preclinical and clinical testing	Modulation of glutamatergic system NMDA-receptor antagonist	Medication for alcohol use disorder	Favorable safety profile Efficacy in treating alcohol use disorder	Dosage 3x per day Contraindicated in severe renal impairment
Memantine	Conflicting	Poor	Further preclinical and clinical testing	Non-competitive NMDA-receptor antagonist	Treatment of Alzheimer disease	Favorable safety and tolerability profile	Minor to no effect on craving in alcohol use disorder trials
Amantadine	Conflicting	Limited	Further preclinical testing and clinical testing in subpopulation with comorbid Parkinson’s disease	Dopamine reuptake inhibitor Non-competitive NMDA-receptor antagonist	Antiparkinsonian agent	Modulation of multiple important neurotransmitter systems Efficacy in treating levodopa-induced dyskinesia	Adverse events Cardiovascular risk
Gabapentinoids	Promising	Poor	Further preclinical and clinical testing	Inhibition of Na+ and Ca2+ channels	Anticonvulsant	Favorable safety profile Renal excretion	Risk of misuse
Modafinil	Conflicting	Limited	Further preclinical and clinical testing	Dopamine reuptake inhibitor	Wakefulness-promoting agent	Promising effect in high impulsivity patients	Potentially harmful in low impulsivity patients
GLP-1 receptor agonists	Promising results in preclinical and clinical trials on substance use disorders and behavioral addictions	No data available on GD	Preclinical and clinical testing	GLP-1 receptor agonist	Antidiabetic	Potential efficacy across multiple addiction types Long-term diabetes and cardiovascular risk reduction	Adverse events (particularly gastrointestinal) Potential risks in normal BMI/underweight patients (loss of lean body mass) High cost
Ketamine/Esketamine	Promising results in clinical trials on substance use disorder Potentially highly promising in comorbid depressive disorder	Poor	Further preclinical and clinical testing	NMDA-receptor antagonist	Anesthetic	Rapid effects on depressive symptoms and neuroplasticity Potential enhancement of psychotherapy efficacy	Risk of misuse

This indicates a clear need for further research efforts in the field of pharmacological treatment, but possibly with a different methodological approach. The high response rate to placebos has been a consistent problem in most studies conducted and makes it difficult to detect clinically significant effects of medications. A placebo lead-in phase has been used in several of the discussed studies with varying degrees of success. The sequential parallel comparison design (SPCD) could be an interesting option, as it has been shown to reduce placebo response rates and improve assay sensitivity in psychiatric studies (Benedetti et al., 2016). This is a variant of the placebo run-in design, which comprises two randomization phases with the aim of retaining participants and increasing statistical power. The selection of participants with high baseline severity in alcohol use disorder also resulted in a lower placebo effect (Scherrer et al., 2021), a method which could also be applied to future GD trials. The Placebo-Control Reminder Script (PCRS) could be another, easily accessible approach: it is a brief interactive procedure that educates participants about factors known to elicit a placebo response and has been shown to reduce placebo response in depression and psychosis trials (Cohen et al., 2021). As already mentioned several times, regular clinical check-ups and outcome assessments likely have a therapeutic effect in themselves (Enck et al., 2005; Enck and Klosterhalfen, 2019b). This effect could be mitigated to a certain extent by training staff and participants to reduce expectations and improve the accuracy of symptom reporting (Evans et al., 2021). Overall, the high placebo response rate remains a major limitation for many drug trials, and it is possible that one of the above methods alone may not be sufficient, requiring a combination of methods in order to reduce the placebo effect.

We consider consistent methodological designs regarding outcome measures to be another important factor in ensuring the reliability and comparability of future studies. PG-YBOCS and G-SAS are two validated and proven scales that have already been used in most studies, and we recommend that these remain standard scores in the future. An improvement of more than 50% in PG-YBOCS and 35% in G-SAS should be used uniformly for defining treatment response, as these values are considered optimal thresholds (Grant et al., 2024). The latest consensus recommendations also emphasize the need for multidimensional assessments, including health-related quality of life, global functioning, psychosocial functioning, and overall physical and mental health and wellbeing, in order to fully capture the impact of GD on the affected individual (Black et al., 2024). By using such multidimensional outcome measures, potential pharmacological effects that go beyond gambling-specific symptoms could also be detected. As of today, there is no single comprehensive scale that covers all aspects of GD.

Since some studies ran out of funds prematurely during participant recruitment, using less stringent inclusion criteria could not only save resources but also represent a more realistic patient group. Furthermore, the previously discussed hypothesis that psychiatric comorbidities play a central role in the treatment of GD should be investigated in more detail. This could prove particularly relevant for disorders such as BD, ADHD or SUDs which share strong associations and overlapping pathomechanisms with GD. Recent findings suggest that over 80% of individuals with GD have at least one comorbid psychiatric disorder, with the highest rates found for SUDs, affective disorders, and anxiety disorders (Galeazzi et al., 2025). Unless specifically addressed, certain comorbid conditions such as anxiety disorders are associated with poorer long-term treatment outcomes, underscoring the importance of an early diagnosis (Wullinger et al., 2023). Pharmacological treatment should be tailored to the patient’s individual psychiatric profile in order to achieve an optimal response. We suggest that future trials do not exclude common psychiatric comorbidities, but rather investigate certain medications in specific patient groups. By conducting smaller studies on specific comorbidities, limited resources could be used in a more targeted manner and allow for urgently needed long-term observation. Unfortunately, most of the studies conducted did not include a follow-up phase, meaning that possible long-term effects of the drugs tested may not have been detected.

Due to continuous research efforts and advances, neurobiological and genetic markers will likely become increasingly relevant for clinical decision-making in the future. Presynaptic dopaminergic dysfunction resulting in hyperdopaminergic states and alterations in dopaminergic transmission have been associated with GD (Pettorruso et al., 2020). Neuroimaging data consistently show dysregulation in reward-related circuits and indicate increased dopamine release in GD, while also revealing some distinct differences from substance-induced neurobiological changes in the brain (Clark et al., 2019). Furthermore, neuroendocrine markers such as lower adiponectin and higher LEAP-2 concentrations were associated with greater severity of gambling addiction and cognitive distortions, providing information and comprehension of the complex neurobiological interactions involved in GD (Mora-Maltas et al., 2024). Genetic polymorphisms in dopamine receptor and neurotrophin family genes have also been linked to an increased susceptibility to GD (Lobo et al., 2015; Solé-Morata et al., 2022). Such findings might have future relevance in individualized treatment planning and predicting response to different medications.

As of today, psychological interventions are considered the primary treatment option of GD. Guidelines recommend cognitive behavioral therapy and motivational interviewing in particular as best practice and first choice. If comorbid psychiatric disorders are present, these should be treated consistently and in parallel to ensure the success of the therapy. In addition to established psychological and pharmacological interventions, other biological forms of therapy (e.g., non-invasive brain stimulation) and digital therapies (e.g., virtual reality exposure therapy, blended online treatment) could be used for patients with GD in the future. By modulating various dysfunctional neurobiological systems, including the dopaminergic system and mesocorticolimbic reward circuits, repetitive transcranial magnetic stimulation (rTMS) can improve executive control and decrease impulsivity (Kan et al., 2023). rTMS, particularly of the left dorsolateral prefrontal cortex, has already been shown to produce long-lasting reductions in gambling cravings (Casula et al., 2025). Another interesting and promising development in the field is psychedelic-assisted therapy (PAT). PAT has successfully been used for treatment of various mental disorders including AUD and other SUDs, with mechanisms of action involving disruption of maladaptive neural circuits and increased executive control over emotional processes (Romero et al., 2024; Meshkat et al., 2025). A RCT on psilocybin-assisted psychotherapy for GD has been proposed, but has not yet been conducted (Jérémie Richard, 2024).
